# Optogenetic Suppression of Lateral Septum Somatostatin Neurons Enhances Hippocampus Cholinergic Theta Oscillations and Local Synchrony

**DOI:** 10.3390/brainsci13010001

**Published:** 2022-12-20

**Authors:** Nelson Espinosa, Alejandra Alonso, Mauricio Caneo, Constanza Moran, Pablo Fuentealba

**Affiliations:** Departamento de Psiquiatria, Centro Interdisciplinario de Neurociencia, Facultad de Medicina, Pontifica Universidad Católica de Chile, Santiago 8331150, Chile

**Keywords:** optogenetics, somatostatin neurons, theta oscillations

## Abstract

The septal complex regulates both motivated and innate behaviors, chiefly by the action of its diverse population of long-range projection neurons. A small population of somatostatin-expressing GABAergic cells in the lateral septum projects deep into subcortical regions, yet on its way it also targets neighboring medial septum neurons that profusely innervate cortical targets by ascending synaptic pathways. Here, we used optogenetic stimulation and extracellular recordings in acutely anesthetized transgenic mice to show that lateral septum somatostatin neurons can disinhibit the cholinergic septo-hippocampal pathway, thus enhancing the amplitude and synchrony of theta oscillations while depressing sharp-wave ripple episodes in the dorsal hippocampus. These results suggest that septal somatostatin cells can recruit ascending cholinergic pathways to promote hippocampal theta oscillations.

## 1. Introduction

Large-amplitude theta oscillations dominate field potential hippocampal activity during active locomotion [1,2] and cognitive processing [3,4], but also during aroused immobility [5] and anxiety [6]. The theta rhythm has been proposed to be essential for sensorimotor integration, temporal coding, and synaptic plasticity [2]. Nonetheless, theta activity is not homogeneous, and two bands have been identified. Indeed, type 1 theta activity is fast (6–12 Hz), atropine-resistant, arises during voluntary or goal-directed motor behavior, whereas type 2 theta activity occurs during immobility, is slower (4–8 Hz), anesthetic-resistant, and sensitive to cholinergic-antagonists [7,8]. While type 1 theta activity seems to depend on inputs from the entorhinal cortex and is associated with spatial encoding and novelty detection, type 2 theta activity requires functional afferents from the CA3 area and is associated with arousal and anxiety [9]. Importantly, both theta bands rely on the integrity of the septal complex, in particular the medial septum, which densely innervates the hippocampus and entrains the theta rhythm [10].

The septal complex is heavily interconnected with the hippocampus [11,12]. While the medial septum provides ascending projections to the hippocampus and prominently contributes to the coordination of theta oscillations and locomotor behavior [13,14], the lateral septum receives descending hippocampal projections and is also essential to regulate locomotion speed and innate behaviors [15]. The main septo-hippocampal projection pathways are GABAergic [16], cholinergic [17], and glutamatergic [18]. Ever since long-range projection GABAergic cells were described, it has been proposed that individual cells exert simultaneous local and distal inhibition [19,20], thus suggesting that this type of neuron is ideally suited to synchronizing local and remote circuits. In fact, the basal forebrain somatostatin cells provide functional inhibitory input to all three long-range neuronal populations [21] and previous work has shown their relevance to regulate excitability in executive centers, such as the prefrontal cortex [22].

Consequently, here, we tested the hypothesis that the functional integrity of lateral septum somatostatin cells is required to sustain the hippocampal theta rhythm. Accordingly, we used optogenetic inhibition of somatostatin cells in the dorsal septum and found that it locally enhanced the septal spiking output, resulting in increased amplitude and synchrony of theta oscillations in the hippocampus. The effect was evident under anesthesia and sensitive to cholinergic antagonists locally applied to the hippocampus, suggesting that, at a minimum, type 2 theta activity was affected by somatostatin cells. We conclude that somatostatin cells can regulate ascending septal pathways to control cortical oscillations and likely modulate associated behaviors.

## 2. Materials and Methods

All procedures involving experimental animals were performed in accordance with Animal Research: Reporting of In Vivo Experiments (ARRIVE) guidelines, reviewed, and approved by university and national bioethics committees. Experiments were carried out with 8- to 30-week-old mice (*n* = 24 from either sex), in accordance with the institutional Ethics Committee (protocol ID 151027003).

**Animals.** Three mice strains from Jackson laboratories were used in this study, C57Bl/6J (stock N° 000664), Ai39 (stock N° 014539, B6, 129S-Gt(ROSA)26Sortm39(CAG-HOP/EYFP)Hze/J), and Sst-IRES-Cre (stock N° 013044, Ssttm2.1(cre)Zjh/J and stock N° 018973, B6N.Cg-Ssttm2.1(cre)Zjh/J). They were used as controls and we refer to them as (Natronomonas pharaonis halorhodopsin) NpHR- animals in the text. Double transgenic animals were obtained from the breeding of Sst-IRES-Cre and Ai39 mice, so that they expressed functional NpHR exclusively in somatostatin cells. We refer to such animals as NpHR+ in the text. Mice were genotyped by PCR on ear biopsies using the primers: GGG CCA GGA GTT AAG GAA GA (common), TCT GAA AGA CTT GCG TTT GG (wild type forward), TGG TTT GTC CAA ACT CAT CAA (mutant forward) for CRE Mice, and CTT TAA GCC TGC CCA GAA GA (wild type reverse), ATA TCC TGC TGG TGG AGT GG (mutant forward), GCC ACG ATA TCC AGG AAA GA (mutant reverse), and TCC CAA AGT CGC TCT GAG (wild type forward) from Integrated DNA Technologies.

**In vivo electrophysiological recordings.** Animals were induced with isoflurane (Baxter Healthcare of Puerto Rico, Guayama, PR, USA), then anesthetized with urethane (Sigma Aldrich, Saint Louis, MO, USA)(0.8 g/kg), and, after 20 min, a dose of ketamine (Richmond Laboratories, Buenos Aires, Argentina) (40 g/kg)/xylazine (Centrovet Ltda., Santiago, Chile) (4 g/kg) to start the surgical procedures. Throughout the experiment 1/12 of the initial dose of urethane was administered every 20–30 min. All drugs were administered intraperitoneally. Rectal temperature was monitored throughout the experiment and was kept at 36 ± 1 °C with a heating pad. Glucosaline solution was injected subcutaneously every 2 h. In fully anesthetized mice, the scalp was cut and retracted to expose the skull. Mice were then implanted with a customized lightweight metal head holder and the head was held in a custom-made metallic holder. Next, small craniotomies (~1 mm) were made with a dental drill above the basal forebrain, to target medial septum (MS; AP 1.0 mm, ML 0.0 mm, DV 2.5 mm from Bregma), and the hippocampus, to target the CA1 region (AP −2.5 mm at an angle of 20°, ML 2 mm, DV 1.2 mm from Bregma). Neuronal activity in MS was recorded by using a 16-channel silicon probe (A1 × 16-Poly2-Std, Neuronexus) stained with DiI and connected to an optic fiber (200 μm in diameter) attached to the shank (optrode), so electrical recording and photostimulation could be achieved simultaneously on the same region. Neuronal activity in the hippocampus was recorded extracellularly with a 32-channel 4-shank silicon probe (Buzsáki 32, Neuronexus, Ann Arbor, MI, USA) (mean resistance 1 MΩ) stained with DiI. Electrical activity was recorded with a 32-channel Intan RHD 2132 amplifier board connected to an RHD2000 evaluation system (Intan Technologies, Los Angeles, CA, USA). Single-unit activity and local field potential (LFP; sampling rate 20 kHz) were digitally filtered between 300 Hz–5 kHz and 0.3 Hz–2 kHz, respectively. Spike shape and amplitude were monitored during recording to ensure that the same cells were recorded.

To allow local drug injection, the hippocampal craniotomy was extended in the ML direction and a 50 μm tip pipette was inserted dorso-ventrally with a 20° angle towards the midline. For the blockade of cholinergic receptors in CA1, 200 nl of atropine (2 mM) and mecamylamine (2 mM) (1:1) (Sigma Aldrich, Saint Louis, MO, USA) were microinjected at 1.2 mm DV (IM-9B microinjector, Narishige, Amityville, NY, USA), at minute 5 of the recording, while giving pulses of light on the MS and recording from hippocampus.

**Optogenetic Stimulation.** Optogenetic stimulation of somatostatin neurons was achieved with a 200 μm optic fiber, (N.A. 0.37, Thorlabs, Newton, NJ, USA) coupled to a green laser (532 nm) that provided a total light power of 0.1–60 mW at the optrode tip. Light stimuli consisted of 5 s light pulses and power at the tip of the fiber was set at 10–15 mW for 200 μm optic fiber. Every recording session lasted 10 min, during which laser stimulation was continuously presented for 5 s every 20 s (i.e., 15 s off–5 s on).

**Histology.** At the end of recordings, mice were terminally anesthetized and intracardially perfused with saline followed by 20 min fixation with 4% paraformaldehyde. Brains were extracted and postfixed in paraformaldehyde for a minimum of 8 h before being transferred to PBS azide and sectioned coronally (60–70 μm slice thickness). Sections were further stained for Nissl substance. Location of shanks and optical fiber were determined referring to standard brain atlas coordinates under a light transmission microscope Nikon DS-Fi2 (Nikon, Melville, NY, USA).

**Spike sorting.** Semiautomatic clustering was performed by KlustaKwik, a custom program written in C++ (https://github.com/kwikteam/klustakwik2/, accessed last on 1 October 2019). This method was applied over the 32 channels of the silicon probe, grouped in eight pseudo-tetrodes of four nearby channels. Spike clusters were considered single units if their auto-correlograms had a 2 ms refractory period and their cross-correlograms with other clusters did not have sharp peaks within 2 ms of 0 lag.

**Unit cross-correlation analysis.** Neural activity in the MS and hippocampus was cross-correlated with the light pulse by applying the “sliding-sweeps” algorithm. A time window of ±15 s was defined with point 0 assigned to the light onset. The timestamps of the hippocampal and basal forebrain spikes within the time window were considered as a template and were represented by a vector of spikes relative to t = 0 s, with a time bin of 500 ms and normalized to the basal firing rate of the neurons. Thus, the central bin of the vector contained the ratio between the number of neural spikes elicited between ±250 ms and the total number of spikes within the template. Next, the window was shifted to successive light pulses throughout the recording session, and an array of recurrences of templates was obtained. Both neural timestamps and start times of light pulses were shuffled by randomized exchange of the original inter-event intervals and the cross-correlation procedure was performed on the random sequence.

**Spectral analysis.** Time–frequency decomposition of LFP was performed with multi-taper Fourier analysis implemented in the Chronux toolbox (http://www.chronux.org, Cold Spring Harbor Laboratory, Cold Spring Harbor, NY, USA, accessed last on 1 October 2019). LFP was downsampled to 500 Hz before decomposition. The same taper parameters described for the coherence analysis were used. To estimate gamma band power, spectra were normalized by 1/f to correct for the power law governing the distribution of EEG signals. To compute power and frequency of the gamma band oscillation, LFP was band-pass filtered with a two-way least squares finite impulse response (FIR) filter (eegfilt.m from the EEGLAB toolbox; http://www.sccn.ucsd.edu/eeglab/, Swartz Center for Computational Neuroscience, La Jolla, CA, USA, accessed last on 1 October 2019).

**Ripple detection.** Sharp-wave ripples were recorded in dorsal CA1, as close as possible to stratum pyramidale. We used a previously described method for ripple detection (Logothetis et al., 2012) with some variation. Briefly, the hippocampus LFP was first down-sampled to 500 Hz, then band-pass filtered (100–250 Hz) using a zero-phase shift non-causal FIR filter with 0.5 Hz roll-off. Next, the signal was rectified, and low-pass filtered at 20 Hz with a 4th order Butterworth filter. This procedure yields a smooth envelope of the filtered signal, which was then z-score normalized using the mean and SD of the whole signal in the time domain. Epochs during which the normalized signal exceeded a 3.5 SD threshold were considered as ripple events. The first point before threshold that reached 1 SD was considered the onset and the first one after threshold to achieve 1 SD as the end of events. The difference between onset and end of events was used to estimate the ripple duration. We introduced a 50 ms refractory window to prevent double detections. To precisely determine the mean frequency, amplitude, and duration of each event, we performed a spectral analysis using Morlet complex wavelets of seven cycles. The Matlab toolbox used is available online as the LAN toolbox (https://bitbucket.org/marcelostockle/lan-toolbox/wiki/Home, accessed last on 1 October 2019).

**Coherence.** Both spike–field and LFP–LFP coherence were computed using the multitaper Fourier analysis and the Chronux toolbox (http://www. chronux.org, Cold Spring Harbor Laboratory, Cold Spring Harbor, NY, USA, accessed last on 1 October 2019). We used 500 data points at 1000 Hz, a time–bandwidth product (TW) of 3 and 5 tapers, resulting in a half width of 0.6 Hz.

**Pairwise phase consistency.** Pairwise phase consistency (PPC, [23]) was computed with ft_connectivity_ppc.m, a Matlab function implemented in Fieldtrip (http://www.fieldtriptoolbox.org/reference/ft_connectivity_ppc, accessed last on 1 October 2019). Briefly, the phase was extracted using the Hilbert transform and the mean of the cosine of the absolute angular distance among all pairs of phases was calculated. This procedure was applied from 0 to 10 Hz (bin = 0.25 Hz).

**Granger causality.** The multivariate Granger causality (MVGC) Matlab toolbox [24] was used to assess pairwise causalities between LFP–LFP and LFP–multiunit activity. This toolbox, available online (https://users.sussex.ac.uk/~lionelb/MVGC/html/mvgchelp.html#1, accessed last on 1 October 2019), allows a fast and accurate estimation of the Wiener–Granger causal inference in the frequency domain. Estimators were calculated with the standard ordinary least squares and with a model order of 50. Frequency resolution was set at 1000.

**Statistics.** Data sets were tested for normality using the Kolmogorov–Smirnov test and then compared with the appropriate test (*t*-test or Wilcoxon two-sided rank sum test). Statistical significance of data for protocols with factorial design (i.e., light on/off conditions) were assessed using two-way repeated-measures ANOVA followed by false discovery rate (FDR) for multiple comparison corrections or Kruskal–Wallis test followed by Mann–Whitney U contrasts.

## 3. Results

### 3.1. Lateral Septum Somatostatin Cells Regulate Firing Patterns in the Medial Septum and Dorsal Hippocampus

**Figure 1:**
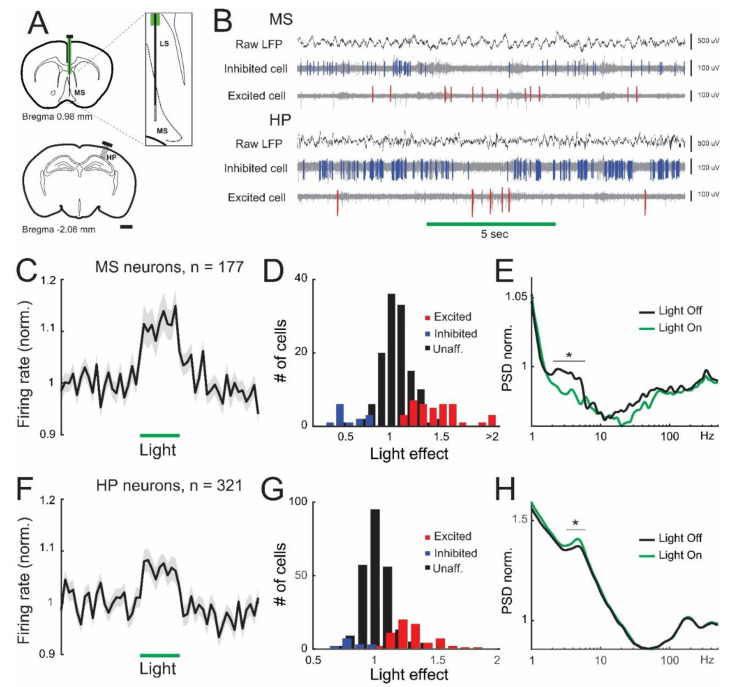
Neuronal activity in the medial septum and dorsal hippocampus during optical inactivation of somatostatin cells in the medial septum. (**A**) Recording locations represented in schematic coronal brain sections. A four-shank multielectrode angled 20° from the midline and an optrode (vertical line) were stereotaxically implanted in the dorsal hippocampus and medial septum, respectively. Scalebar: 1 mm. Inset, optic fiber (green) and multielectrode (black) targeted lateral septum (LS) and medial septum (MS), respectively. (**B**) Example simultaneous electrophysiological recordings (mouse NE115, recording 01) showing LFP and multiunit activity (LFP filtered 500 Hz−5 kHz) from the medial septum (MS, channels 39, 48, and 34, respectively) and dorsal hippocampus (HP, channels 20, 16, and 25, respectively). Horizontal bar depicts laser stimulation (5 s, 10 mW fiber diameter 200 um) and colors depict inhibited (blue events) and excited (red events) units. (**C**) Average normalized discharge probability for all septal neurons (mean value ± SEM, *n* = 177 units, 7 animals). Bin size: 500 ms. (**D**) Distribution during light stimulation for septal neurons. Blue and red bars denote statistical difference from shuffled data. (**E**) Averaged power spectral density (PSD) for all recorded neurons before (black line) and during light stimulation (green line). Asterisk in band 2–6 Hz: paired *t*-test, *p* = 0.016. (**F**) Average normalized discharge probability for all hippocampal neurons (mean value ± SEM, *n* = 321 units, 7 animals). Bin size: 500 ms. (**G**) Distribution during septal stimulation for hippocampal neurons. Blue and red bars denote statistical difference from shuffled data. (**H**) Averaged power spectral density (PSD) for all recorded neurons before (black line) and during light stimulation (green line). Asterisk in band 4–7 Hz: paired *t*-test, *p* = 0.0003.

We used transgenic mice selectively expressing YFP-labeled halorhodopsin (NpHR) in somatostatin cells (Figure S1) [22]. We first established the relevance of somatostatin cells for the spontaneous activity patterns in the medial septum. For this, we stereotaxically implanted a silicon probe in the medial septum of urethane-anesthetized transgenic animals, coupled with an optic fiber on the dorsal septum (Figure 1A). As previously reported, we used extended laser pulses to achieve maximal inhibition of somatostatin cells and reproduce prior experimental protocols [21,22,25]. In doing so, we obtained diverse types of response in individual septal units (Figure 1B), yet the net effect of optogenetic stimulation on the medial septum was excitation, as the global firing rate increased by 12.7% on average (Figure 1C). Indeed, upon optogenetic stimulation, a small fraction of septal cells (7.3%, *n* = 13 units) strongly decreased their activity (by 45.5 ± 4.3%), whereas another minor neuronal population (21.5%, *n* = 38 units) increased its firing rate (by 53.2 ± 7.9 %), presumably by synaptic disinhibition (Figure 1D, Figure S1). Baseline firing rates and spike waveforms were very similar between excited and inhibited units (Figure S2), consistent with previous results in the basal forebrain [22]. Next, we estimated whether the increased neuronal discharge was associated with specific frequency bands of activity. For this, we computed the power of neuronal activity across the frequency spectrum. A fraction of medial septum units exhibited preference to discharge in the low theta frequency band (2–6 Hz). Interestingly, such a tendency was selectively abolished upon optogenetic suppression of somatostatin cells (*p* = 0.016, Figure 1E). We also assessed the effect of the medial septum’s synaptic output on dorsal hippocampal targets. Indeed, our medial septum recordings were coupled with simultaneous monitoring of dorsal hippocampal activity (Figure 1A), where single units exhibited mixed results upon optogenetic stimulation in the dorsal septum (Figure 1B). The overall effect of lateral septum optogenetic stimulation in the dorsal hippocampus was excitation, as the global firing rate increased by 8.4% (Figure 1F). Inhibition of septal somatostatin cells robustly affected hippocampal spiking activity, with a significant proportion of hippocampal neurons (20.2%, *n* = 65) consistently increasing their firing rate (by 34.1 ± 3.4%), whereas a minor proportion of hippocampal units (4.7%, *n* = 15) was inhibited (by 19.4 ± 3.3%) by laser stimulation. Laser-excited hippocampal units had low spontaneous firing rates, broad spike waveforms, and were strongly coupled to sharp-wave ripples (Figure S2), consistent with the characteristics of pyramidal cells [25]. Conversely, laser-inhibited hippocampal cells showed high spontaneous firing rates, narrow spikes, and were moderately coupled to sharp-wave ripples (Figure S2), consistent with the reported attributes of interneurons [25]. Thus, our results suggest that optogenetic suppression of septal somatostatin neurons disinhibits pyramidal cells in the hippocampus, likely by decreasing interneuron activity (Figure 1G). Spiking activity of hippocampal units exhibited bimodal preference in the frequency spectrum, with prominent peaks in the theta band (3–7 Hz) and the fast-frequency range (200 Hz, Figure 1H), likely resulting from bursting discharges of pyramidal cells (Figure S2). Interestingly, optogenetic inhibition of septum somatostatin cells selectively increased the amplitude of theta-frequency discharge in hippocampal units, without affecting the fast-frequency range (*p* = 3 × 10^−4^, Figure 1H). Thus, lateral septum somatostatin cells can regulate the activity of local and distal synaptic targets, differentially affecting neuronal firing rates and spectral frequency preferences, particularly in the theta range.

### 3.2. Inhibition of Lateral Septum Somatostatin Cells Enhances Theta Oscillations in the Dorsal Hippocampus

**Figure 2:**
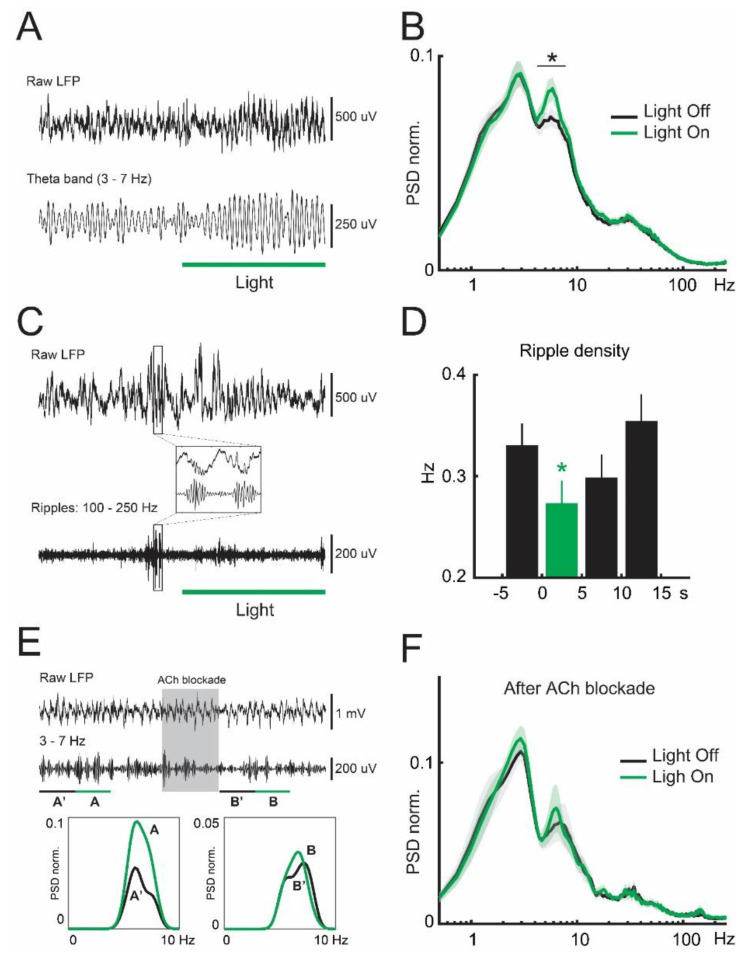
Theta oscillations and ripple density in the dorsal hippocampus during septal somatostatin cells inhibition. (**A**) Raw LFP (top panel), and theta-band-filtered LFP (3−7 Hz, bottom panel) for an example electrophysiological recording in the dorsal hippocampus (mouse NE113, recording 05, channel 29). (**B**) Average normalized power spectral density (PSD) of the dorsal hippocampal LFP in the presence (green line) or absence (black line) of optogenetic stimulation. Asterisk in band 4–7 Hz: paired *t*-test, *p* = 7.99 × 10^−7^, *n* = 27 recordings. (**C**) Raw LFP (top panel), and ripple-band LFP (100−250 Hz, bottom panel) for an example electrophysiological recording in the dorsal hippocampus (mouse NE113, recording 05, channel 29). Inset, example sharp-wave ripple episodes before light stimulation. (**D**) Inhibition of septal somatostatin cells decreases ripple density (green bar, mean value ± SEM, bin = 5 s). Asterisk: one-way ANOVA test, and Bonferroni post-hoc correction, *p* = 0.0131, *n* = 262 trials. (**E**) Example electrophysiological recording (mouse NE125, recording 03, channel 20) showing response patterns before and after drug administration (gray box). From top to bottom: raw LFP, theta-band-filtered LFP, and PSD during control (black line) and optogenetic stimulation (green line). (**F**) Average normalized power spectral density (PSD) of the dorsal hippocampal LFP after cholinergic receptors blockade.

We then studied the field potential effects of septal optogenetic stimulation. Inhibition of somatostatin cells did not show evident changes in the local field potential of the medial septum (Figure S3). Conversely, optogenetic stimulation selectively increased the power of theta oscillations in the dorsal hippocampus (Figure 2A) by 14.8% (*p* = 8 × 10^−7^, Figure 2B), with no noticeable effects on other prominent rhythms such as delta or gamma oscillations (Figure 2B, Table 1). In addition, silencing somatostatin cells significantly decreased the density of sharp-wave ripples (Figure 2C) by 17.3% (*p* = 0.013, Figure 2D). These results are consistent with the selective activation of ascending cholinergic transmission from the medial septum [26,27]. To further test this idea, we locally applied cholinergic receptor antagonists in the dorsal hippocampus during optogenetic inhibition of septal somatostatin cells (Figure 2E). Cholinergic blockers transiently abolished the enhancement of hippocampal theta oscillations (Figure 2F), an effect specific to that frequency band (*p* = 0.259), as it did not affect other cortical rhythms (Figure 2F, Table 2). While these experiments do not exclude the involvement of GABAergic and glutamatergic transmission, they suggest that enhanced hippocampal theta oscillations during optogenetic inhibition of septal somatostatin cells were largely mediated by cholinergic transmission in the hippocampus.

**Figure 3:**
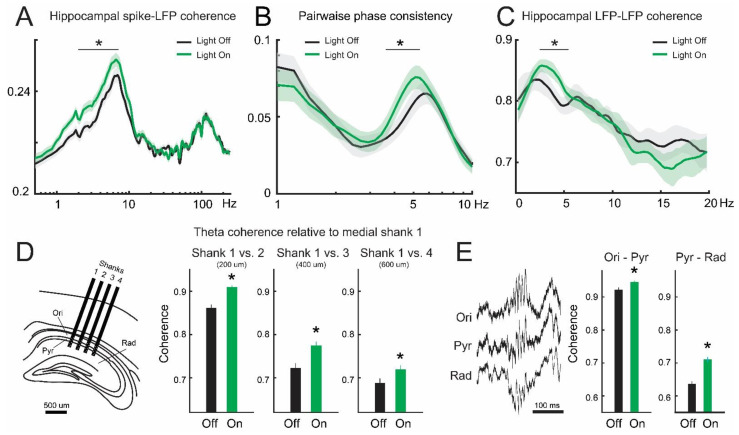
Intrahippocampal synchrony during optogenetic inhibition of septal somatostatin cells. (**A**) Average spike-field coherence between dorsal hippocampal units and LFP pyramidal layer before (off, black line, 5 s) and during light stimulation (on, green line, 5 s). Asterisk in band 2–7 Hz: W = 16,976, *p* = 9.9 × 10^−8^, Wilcoxon signed-rank test, *n* = 321 cells. (**B**) Average pairwise phase consistency (PPC) in the dorsal hippocampus. Wilcoxon signed-rank test in band 4–6 Hz. W = 21,510, * *p* = 0.0189, *n* = 321 cells. (**C**) Average LFP–LFP coherence around the pyramidal layer (i.e., between the more dorsal and ventral channels of the shank). Asterisk in band 3–6 Hz: paired *t*-test, *p* = 0.00016, *n* = 27 recordings. (**D**) Statistically significant increase in hippocampal theta coherence across the medio-lateral axis, where magnitude is independent of the distance between shanks (two-way ANOVA, *p* < 10^−6^). (**E**) Example sharp-wave ripples recorded at different depths in the dorsal CA1 (left) and average LFP–LFP coherence around the pyramidal layer (right). The theta-promoting effect of septal stimulation was segregated between hippocampal layers, being more prominent between the stratum radiatum and pyramidal layer. Stratum oriens–stratum pyramidale coherence: Wilcoxon signed-rank test, W = 2904, * *p* = 2.24 × 10^−5^, *n* = 140 trials. Stratum pyramidale–stratum radiatum: Wilcoxon signed-rank test, W = 1213, * *p* = 3.65 × 10^−29^, *n* = 210 trials.

Previous reports have shown that enhanced theta oscillations in the dorsal hippocampus increase local synchrony [26]. Hence, we tested this option for both field potentials and single-neuron activity. We studied the coordination of single units and field potentials in the hippocampus. Hippocampal cells also increased their coupling with theta oscillations during optogenetic stimulation, as evidenced by both enhanced spike-field coherence (*p* = 9.9 × 10^−8^, Figure 3A) and the unbiased pairwise phase consistency (PPC, Figure 3B). Similarly, global intrahippocampal synchrony of theta oscillations increased during optogenetic stimulation, as evidenced by enhanced coherence of field potentials, particularly for theta oscillations (*p* = 1.6 × 10^−4^, Figure 3C). Moreover, theta synchrony augmented in both the dorsoventral and mediolateral axes. Indeed, the coordination of theta oscillations compared between shanks of the silicon probe was consistently larger during optogenetic inhibition of septal somatostatin cells (Figure 3D). Further, by using the polarity and shape of sharp-wave ripples we were able to infer the location of recording electrodes across the silicon probe [28]. Hence, we compared theta synchrony across the dorsal CA1 layers. We found that theta coherence significantly increased between stratum pyramidale and its neighboring layers when lateral septum somatostatin cells were inhibited (Figure 3E). Hence, our results confirm that enhanced theta oscillations in the dorsal hippocampus increase local synchrony between both single neurons and field potentials.

### 3.3. Lateral Septum Somatostatin Cells Modulate Functional Connectivity between Medial Septum and Dorsal Hippocampus during Theta Oscillations

**Figure 4:**
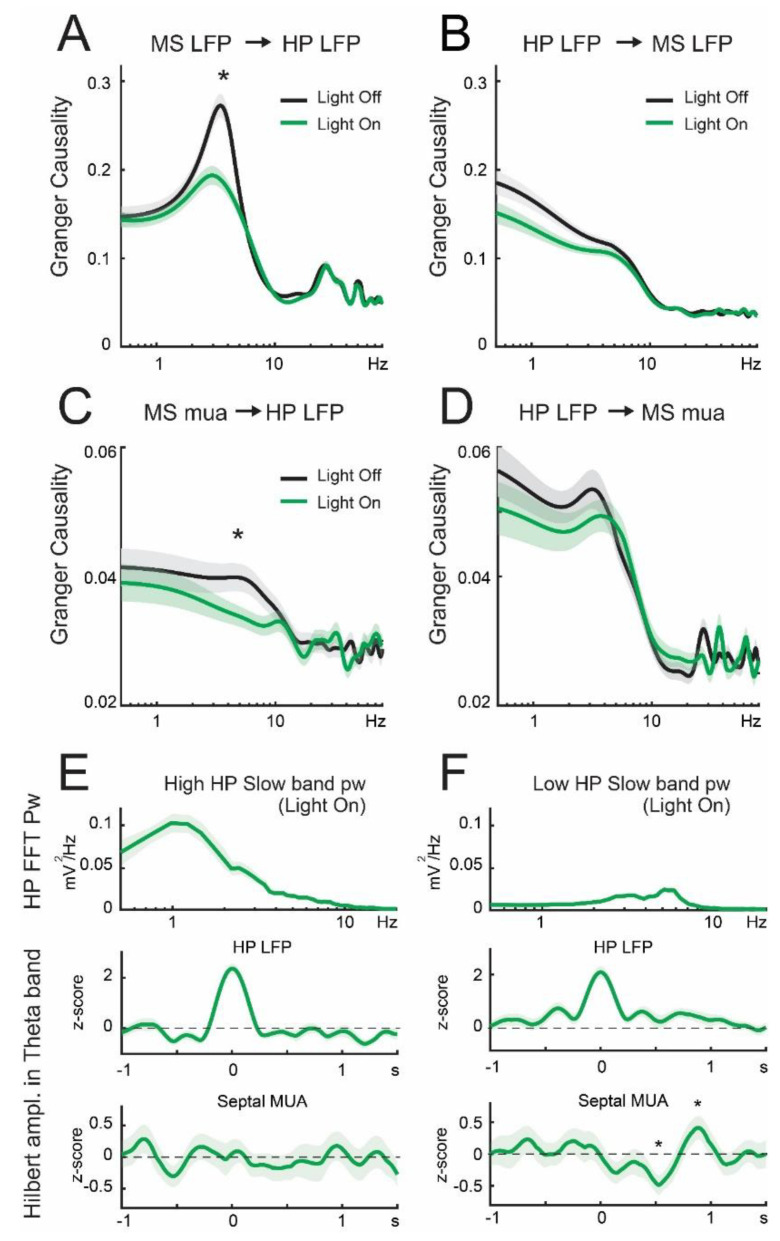
Granger causality between medial septum and dorsal hippocampus during optical inactivation of septal somatostatin cells. (**A**) Averaged Granger causality from the septal LFP to hippocampal LFP (MS LFP -> HP LFP). Septal somatostatin cell inhibition statistically decreases the peak frequency. Asterisk in band 3–6 Hz: W = 52,226, *p* = 2 × 10^−6^, Wilcoxon signed-rank test, *n* = 405 trials. (**B**) Reverse Granger causality (HP LF -> MS LFP) is unaffected by light stimulation. (**C**) MS -> HP connectivity in theta band was weakened by inhibition of septal somatostatin neurons. Asterisk in band 3–7 Hz: paired *t*-test, *p* = 0.0143, *n* = 405 trials. (**D**) HP -> MS connectivity is apparently unaffected by optogenetic stimulation. (**E**) Average values for the 5th lower percentile in Granger causality HP LFP -> MS MUA and 5th upper percentile in hippocampal slow wave power (*n* = 19 trials) of trials during light stimulation. Top panel: average normalized power spectral density of hippocampal LFP. Middle panel: z-scored theta-filtered amplitude for hippocampal LFP averaged with peak value set at t = 0. Bottom panel: average z-scored theta-filtered amplitude for septal LFP and referred to peak value of hippocampal theta. (**F**) Average values for the 5th upper percentile in Granger causality HP LFP -> MS MUA and 5th lower percentile in hippocampal slow wave power (*n* = 19 trials) of trials during light stimulation. Top panel: average normalized power spectral density of hippocampal LFP. Middle panel: z-scored theta-filtered amplitude for hippocampal LFP averaged with peak value set at t = 0. Bottom panel: average z-scored theta-filtered amplitude for septal LFP and referred to peak value of hippocampal theta. Asterisk denotes significant difference against zero (Wilcoxon signed-rank test, FDR correction, *p* < 0.0142).

Next, we investigated the directionality of the functional relationship between the medial septum and dorsal hippocampus. To assess directionality, we used Granger causality within the theta frequency band [13]. We found significant, unidirectional connectivity from the septum to the hippocampus (MS -> HP) in the low theta frequency band (3–6 Hz, Figure 4A). Importantly, the MS -> HP connectivity was strongly dependent on the activity of somatostatin cells, as their optogenetic suppression significantly reduced the causality (*p* = 2.4 × 10^−6^, Figure 4A). Reduction in Granger causality was specific to the theta band, as it did not affect other rhythms, such as gamma oscillations (Figure 4A, Table 3). Moreover, the reciprocal connectivity, HP -> MS, did not show apparent relevance for theta band synchrony, neither was it dependent on the activity of lateral septum somatostatin neurons (*p* = 0.078, Figure 4B). We further analyzed multiunit activity (MUA) from the medial septum and found similar patterns in which unidirectional connectivity from the medial septum to the dorsal hippocampus was dependent on the activity of somatostatin cells (Figure 4C). Consistent with the previous observation, the reciprocal connectivity, HP -> MS, was not significant for theta band synchrony, neither was it dependent on the activity of lateral septum somatostatin neurons (Figure 4D). Previous reports have shown that as theta power increases during activated brain states and complementary delta waves decrease, theta Granger causality in the HP -> MS direction selectively increases, but not in the reverse MS -> HP direction [13]. Hence, we tested whether the amplitude of slow oscillations was inversely proportional to theta causality in the HP -> MS direction in our recordings. We found no significant correlation during baseline conditions (*p* = 0.08), yet upon optogenetic suppression of somatostatin cells it became apparent (Spearman’s R = −0.17, *p* = 8 × 10^−4^). Indeed, high power in hippocampal delta waves was associated with low Granger causality in the theta band (Figure 4E), whereas low power in the delta frequency band was related to statistically significant theta Granger causality (Figure 4F). Hence, suppressing somatostatin cells enhances cholinergic theta oscillations in the dorsal hippocampus and regulates the directionality of connectivity between the medial septum and dorsal hippocampus.

## 4. Discussion

We have shown that optogenetic inhibition of somatostatin cells in the dorsal septum locally enhances spiking output, including septo-hippocampal cells, thus resulting in increased amplitude of theta oscillations and decreased incidence of ripple episodes in the dorsal hippocampus. This effect involves the disinhibition of septal cholinergic pathways, as it was blocked by cholinergic antagonists locally applied to the hippocampus. Moreover, functional connectivity in the theta band was causal in the septo-hippocampal direction, and dependent on the activity of somatostatin cells. Our results show that somatostatin cells can regulate ascending septal pathways to promote cortical theta oscillations, which will likely modulate innate and motivated behaviors that are distinctively mediated by subcortical circuits.

In our experiments, laser inhibition of dorsal septum somatostatin cells produced net increased spiking activity in the medial septum, likely due to synaptic disinhibition [29]. The neuroanatomical organization of the septal complex supports this interpretation as there are three main septo-hippocampal projection pathways conveyed by GABAergic [16], cholinergic [17], and glutamatergic [18] neurons, and basal forebrain somatostatin cells provide functional inhibitory input to all those neuronal populations [21,30]. Hence, those cell types are available to elevate their baseline activity levels, and become more sensitive to incoming synaptic barrages, upon optogenetic removal of the inhibitory drive supplied by somatostatin cells. Consequently, distal cortical targets, such as the dorsal hippocampus, were also activated after suppression of septal somatostatin cells. In both the medial septum and dorsal hippocampus, septal laser stimulation produced net excitation, as evidenced by the significant increase in overall firing rates. Nonetheless, the preferred spectral frequency of neuronal discharge was inversely influenced. Indeed, during laser stimulation, hippocampal units increased their activity in the theta band, while septal units reduced their preference to discharge in the theta frequency. The theta discharge of medial septum cells was broader and slower than that of dorsal hippocampus units, consistent with the slow cholinergic theta activity associated with motor behavior originating from the subcortical nucleus, as compared to hippocampal theta oscillations, which also integrate faster noncholinergic components [31,32].

Direct optogenetic activation of septal cholinergic cells excites hippocampal interneurons, which, in turn, inhibit pyramidal cells [27]. Conversely, our septal laser stimulation seemed to produce opposite effects in hippocampal units, with enhanced putative pyramidal cell activity and decreased spiking in putative interneurons. This discrepancy may result from the differential connectivity between cholinergic cells and somatostatin neurons [33]. For example, somatostatin cells are presynaptic to cholinergic neurons in the basal forebrain, and their respective activation produces opposite effects in the sleep–wake cycle [21]. Similarly, silencing somatostatin cells or activating cholinergic cells in the septal complex seems to be sufficient to enhance theta oscillations in the dorsal hippocampus. Type 1 theta activity is faster (6–12 Hz) and arises during voluntary or goal-directed motor behavior, whereas type 2 theta activity is slower (4–8 Hz), occurs during immobility, is resistant to most anesthetics, and is sensitive to cholinergic-antagonists [6,34,35,36]. Hence, our evidence suggests that somatostatin cells participate, at least, in the regulation of hippocampal type 2 theta oscillations.

Further, spectral frequency preferences of individual unitary activity were mirrored in the field potential, as optogenetic inhibition of somatostatin cells significantly increased the power of theta oscillations in the dorsal hippocampus. The local application of cholinergic receptor antagonists confirmed that septo-hippocampal cholinergic pathways were largely responsible for the enhanced hippocampal theta waves [37]. We have previously shown comparable results in the ascending basal forebrain projections innervating the medial prefrontal cortex [22]. Thus, we propose that the transient suppression of GABAergic input provided by somatostatin cells disinhibits septo-hippocampal cells, thus increasing the acetylcholine release on the dorsal hippocampus and consequently enhancing the power and synchrony of theta oscillations [38]. Indeed, similar results were obtained in previous studies, using either patterned optogenetic stimulation of cholinergic septal neurons in vivo [26,27] or bath applications of cholinergic agonists in hippocampus slices [39,40]. Consistent with those reports, the action of acetylcholine in our study was not dependent on a rhythmic input, since septal units decreased their discharge preference in the theta range during laser stimulation and the prolonged laser pulse did not entrain any particular rhythm.

It has been shown that the direct activation of septal cholinergic somata with laser trains under urethane anesthesia increases the power and synchrony of theta oscillations in the hippocampus [26,27]. The increased theta power arises partly from the suppression of peri-theta frequencies, thus resulting in an enhanced signal-to-noise ratio for the theta band. Conversely, we did not detect such changes in the peri-theta frequency bands during inhibition of somatostatin cells, but a very selective increase in the power of the theta band itself. In addition, previous results have shown that optogenetic stimulation of septal cholinergic cells dramatically suppressed ripple episodes in the hippocampus [26,27], a result that we could not attain with the silencing of septal somatostatin cells, as ripple episodes were only partially, yet significantly, decreased. Suppression of sharp-wave ripples might result from the direct activation of interneurons in the CA3 field [27]. Importantly, it has been proposed that cholinergic activation of theta oscillations and suppression of sharp-wave ripples rely on anatomically distinct pathways, with the indirect pathway being responsible for theta enhancement, and the direct pathway, which suppresses sharp-wave ripples [27]. In that case, our results would be better explained by the preferential activation of the indirect pathway, which boosts theta oscillations, and consistent with the differential connectivity between cholinergic and somatostatin neuronal populations.

During examination of the causal interactions between the medial septum and dorsal hippocampus, we found a significant unidirectional influence of the septum over the hippocampus. Urethane anesthesia induces prominent cortical slow waves, so it has been used as a model system to study sleep slow oscillations [41,42]. During slow-wave sleep, there is prominent unilateral causality in the septum to hippocampus direction in the theta frequency band [13], which is similar to the interaction that we describe here under urethane anesthesia. Indeed, we found little descending influence from the hippocampus to the septum, but a prominent peak in theta activity in the opposite direction. Interestingly, despite the prominent theta peak in Granger causality, neither during slow wave sleep [13] nor during our urethane-anesthesia experiments were theta oscillations evident in hippocampus field recordings. This observation has been interpreted as resulting from the little descending theta drive in the hippocampus to septum direction, which is likely necessary to reveal theta oscillations in the field potential [13]. Furthermore, intraseptal injections of somatostatin or the somatostatin receptor agonist octreotide in freely moving rats reduced the power of hippocampal EEG in the theta band [43], thus confirming that somatostatin cells participate in the control of hippocampal theta oscillations. Those results complement our findings, as they suggest that during somatostatin cell activation, hippocampal theta waves are depressed, while our experiments show that during somatostatin cell inhibition, hippocampal theta oscillations are enhanced.

Arousal, locomotion, and theta oscillations are commonly concurrent, yet their mechanisms can be dissociated and independently controlled [44,45,46]. Importantly, the medial septum seems to be a neural hub for the regulation of all these processes. For example, it has recently been shown that medial septum glutamatergic neurons can disinhibit pyramidal cells in the hippocampus and control both theta oscillations and locomotion speed [45]. Hence, goal-directed behavior, such as memory-guided spatial navigation, might be influenced by the action of somatostatin cells and their enhancement of theta oscillations in the dorsal hippocampus. On the other hand, septal somatostatin cells innervate the main local neuronal populations, yet they also express prominent descending projections reaching subcortical targets, such as the lateral hypothalamus [47], that have been shown to control feeding preferences [48] and fear conditioning behavior [49]. Indeed, septal somatostatin neurons can gate mobility to calibrate context-specific fear behavior, with descending contextual information provided by the CA3 area [49]. Importantly, septal somatostatin cells produce terminal fields that selectively target the lateral hypothalamus, where a distinct population of glutamatergic neurons powerfully controls the generation of repetitive self-grooming [50]. Additionally, descending theta oscillations through the hippocampo-septal pathway regulate the speed of locomotion. Indeed, theta-patterned stimulation of lateral septum GABAergic projections to the lateral hypothalamus is sufficient to decrease ambulatory behavior [15]. Overall, our results suggest that lateral septum somatostatin cells will be implicated in the control of both motivated and innate behaviors [51].

Interestingly, human studies have confirmed a role for hippocampal theta oscillations in both spatial cognition and anxiety behavior [52,53]. Moreover, while spatial navigation was associated with faster theta waves (4–8 Hz), threat-related anxiety was related to slower theta waves (2–6 Hz). Such distinction in frequency bands is reminiscent of the type 1 and 2 rodent theta bands [54]. In recent years, a quantitative model based on the concept of velocity-controlled oscillators has been proposed for the septo-hippocampal circuit [55], in which type 1 and type 2 theta oscillations determine distinct aspects of the overall theta frequency [56]. Interestingly, despite their neurochemical differences, anxiolytic drugs reduce the frequency of hippocampal theta oscillations during immobility [6]. Conversely, studies in rodents have shown that the power of theta oscillations, particularly in the ventral hippocampus, increases during anxiety-like behavior [57]. These results suggest a tight relation between hippocampal theta oscillations and anxiety [58]. Our results show that regulating activity levels of septal somatostatin cells can affect theta oscillations, with likely consequences for behavior. For example, a recent report has shown that lateral septum somatostatin cells regulate depressive-like behaviors in mice. It is probable that such effects are, at least partly, mediated by cholinergic neurotransmission in the dorsal hippocampus. Hence, we suggest that future investigations should consider septal somatostatin cells as a potential target for the control of altered innate behaviors in translational neuroscience [59].

## Figures and Tables

**Table 1:** Changes induced by light in hippocampal LFP rhythms.

Frequency Band	Light off	Light on	*p*
Slow band (0.5–3 Hz)	73.2 ± 3.3	71.9 ± 3.4	0.531
Theta (4–7 Hz)	68.3 ± 3.3	78.4 ± 4.4	7.99 × 10^−7^ (*)
Slow-gamma (20–40 Hz)	22.5 ± 1.9	23.1 ± 1.8	0.266
High-gamma (60–140 Hz)	6.6 ± 0.5	6.8 ± 0.5	0.158

Values in mean ± SEM 10^−3^ × (uV^2^/Hz). *p* = paired *t*-test, asterisk denotes statistical significance.

**Table 2:** Changes induced by light in hippocampal LFP rhythms under ACh receptors blockade.

Frequency Band	Light off	Light on	*p*
Slow band (0.5–3 Hz)	79.4 ± 7.3	81.8 ± 4.3	0.63
Theta (4–7 Hz)	58.8 ± 9.7	63.1 ± 10.6	0.259
Slow-gamma (20–40 Hz)	17.7 ± 2.7	17.3 ± 2.6	0.784
High-gamma (60–140 Hz)	6.1 ± 1.1	6.3 ± 1.0	0.605

Values in mean ± SEM 10^−3^ × (uV^2^/Hz). *p* = paired *t*-test.

**Table 3:** Changes induced by light on Granger causality from the septal LFP to hippocampal LFP (MS LFP -> HP LFP).

Frequency Band	Light off	Light on	*p*
Slow band (0.5–3 Hz)	0.16 ± 0.009	0.15 ± 0.008	0.97
Theta (3–6 Hz)	0.18 ± 0.008	0.15 ± 0.007	2.4 × 10^−6^ (*)
Slow-gamma (20–40 Hz)	0.08 ± 0.003	0.075 ± 0.003	0.27
High-gamma (60–140 Hz)	0.06 ± 0.001	0.06 ± 0.001	0.74

Values in mean ± SEM. *p* = Wilcoxon signed-rank test. Asterisk denotes statistical significance.

## Data Availability

The data presented in this study are available on reasonable request from the corresponding author. The data are not publicly available yet, but will be in the near future.

## Supplementary Materials

The following supporting information can be downloaded at: https://www.mdpi.com/article/10.3390/brainsci13010001/s1, Figure S1: Anatomical location of recording sites.; Figure S2: Neuronal activity in the medial septum and dorsal hippocampus during optical inhibition of septal somatostatin cells. Figure S3: Medial septum field potential during optogenetic stimulation.
