# Supplementary material for: Optogenetic Suppression of Lateral Septum Somatostatin Neurons Enhances Hippocampus Cholinergic Theta Oscillations and Local Synchrony
Source: Brain Sci. 2022 Dec 20;13(1):1. doi: 10.3390/brainsci13010001 (PMC9856160; doi:10.3390/brainsci13010001)
Supplement: Supplementary file 1 [file brainsci-13-00001-s001.zip › brainsci-2091465-supplementary.pdf]

Supplementary Material

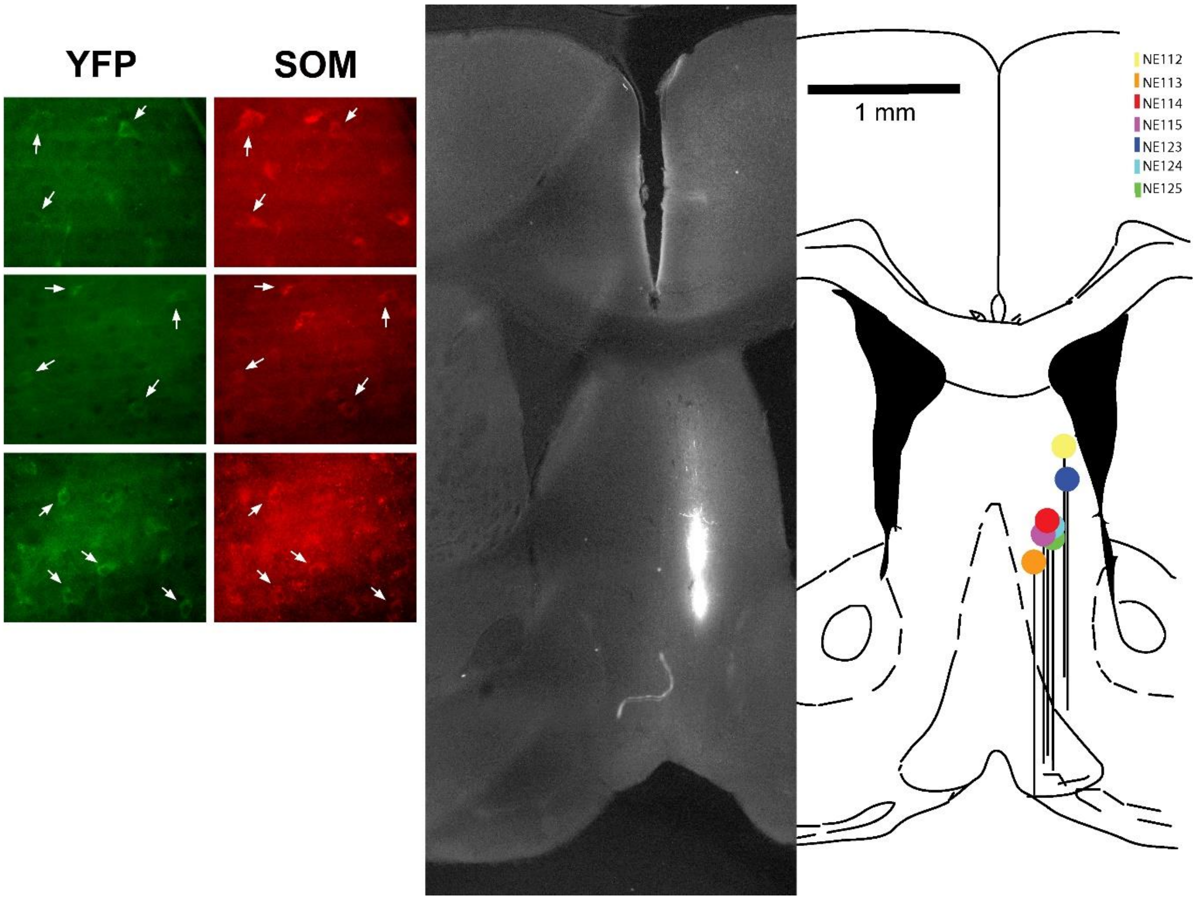

Figure S1. Anatomical location of recording sites. Left, example fluorescent micrographs showing the expression of somatostatin (red) in the dorsal septum of YFP-NpHR+ (green) animals. Middle, example fluorescence microscopy image marking the placement of the tip of the fiber (NE 113). Right, representation of all experiments and placement of the tip of the fiber (colored circles) and the projection of the probe (black horizontal lines). Scale bar 1mm. Antero-posterior 0.98mm in reference to standard atlas coordinates.

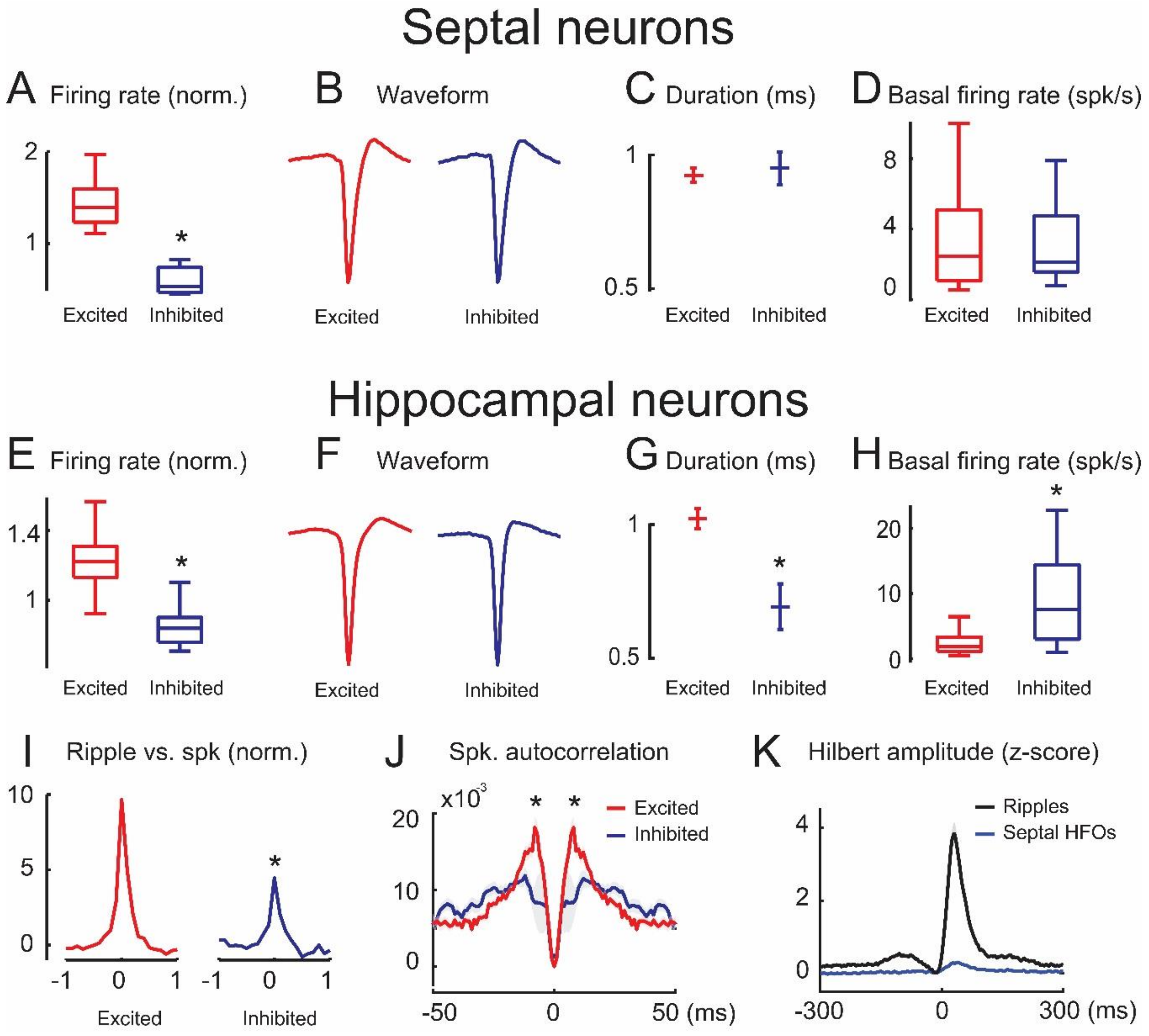

Figure S2. Neuronal activity in the medial septum and dorsal hippocampus during optical inhibition of septal somatostatin cells. Septal neurons: **(A)** normalized discharge probability during optical stimulation for excited (red line, n = 38) and inhibited (blue line, n = 13) neurons (Wilcoxon rank-sum test, $^*P = 9.94\times10^{-8}$). **(B)** spike waveform average. **(C)** waveform peak-to-valley duration (Wilcoxon rank-sum test, P = 0.44). **(D)** basal firing rate (Wilcoxon rank-sum test, P = 0.47).

Hippocampal neurons: **(E)** normalized discharge probability during optical stimulation for excited (red line, n = 65) and inhibited (blue line, n = 15) neurons (Wilcoxon rank-sum test, $^*P = 1.94\times10^{-9}$). **(F)** spike waveform average. **(G)** waveform peak-to-valley duration (Wilcoxon rank-sum test, *P = 0.018). **(H)** basal firing rate (Wilcoxon rank-sum test, *P = 8.63×10−4). **(I)** crosscorrelogram of spiking activity related to hippocampal sharp wave ripples onset (unpaired t-test, *P = 0.0049). **(J)** spiking activity autocorrelation (Wilcoxon rank-sum test, *P = 0.032). **(K)** Ripple-triggered average between the ripple onset (reference) and the Hilbert amplitude for hippocampal LFP (100 – 250 Hz) and high frequency oscillations (HFOs) for Septal LFP (100 – 180 Hz), n = 18 recordings.

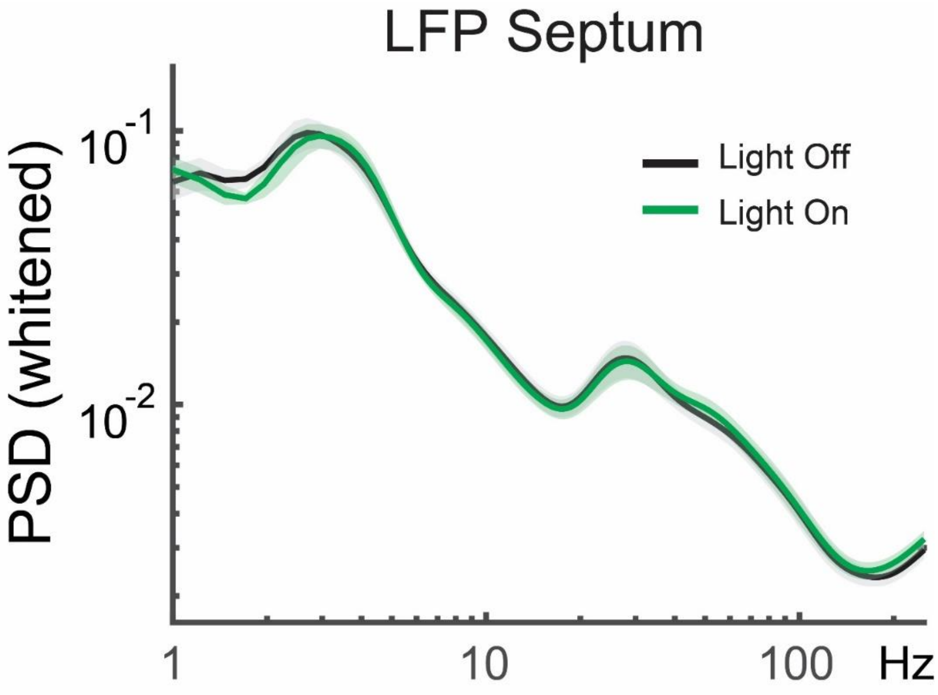

Figure S3. Average normalized power spectral density (PSD) of the medial septum LFP before (Control, black line, 5s) and during light stimulation (Light, green line, 5s).
